# A Prospective, Double-Blind Evaluation of Anterior Cruciate Ligament Reconstruction With Tibialis Tendon Allograft: Donor Age Does Not Alter Outcomes

**DOI:** 10.1016/j.asmr.2022.11.025

**Published:** 2022-12-20

**Authors:** Thomas Carter, Amy Norton

**Affiliations:** Banner University of Arizona-Phoenix, Phoenix, Arizona, U.S.A.

## Abstract

**Purpose:**

To evaluate the effect of graft donor age on outcomes of anterior cruciate ligament (ACL) reconstruction with nonirradiated, fresh-frozen tibialis tendon allografts.

**Methods:**

This prospective, randomized, double-blind, single surgeon, 2-year follow-up study enrolled 40 patients (28 female, 12 male who underwent ACL reconstruction with tibialis tendon allografts. Results were compared with historical outcomes for allografts from donors aged 18 to 70 years. Analysis was determined by Group A (<50 years) and Group B (>50 years). Objective and subjective International Knee Documentation Committee (IKDC) forms, KT-1000 testing, and Lysholm scores were used for the evaluation.

**Results:**

Follow-up on average of 24 months was completed in 37 patients (92.5%; Group A = 17, Group B = 20). Average patient age at surgery for Group A was 42.1 years (range 27-54) and Group B was 41.7 years (range 24-56). None of the patients required additional surgery during the initial 2-year follow-up. At 2-year follow-up, there were no significant differences in subjective outcomes. IKDC objective ratings for Group A were A-15 and B-2, and Group B were A-19 and B-1 (*P* = .45). Average IKDC subjective scores for Group A were 86.1 (± 16.2) and Group B were 84.1 (± 15.6) (*P* = .70). KT-1000 side to side differences for Group A were 0-4, 1-10, and 2-2, and Group B were 0-2, 1-10, 2-6 (*P* = .28). Average Lysholm scores for Group A were 91.4 (± 16.7) and Group B were 88.1 (± 12.3) (*P* = .49).

**Conclusions:**

Donor age was not associated with clinical outcomes after ACL reconstruction using nonirradiated, fresh-frozen tibialis tendon allografts.

**Level of Evidence:**

II, prospective prognostic trial.

It is common practice to reconstruct a torn anterior cruciate ligament (ACL) in an effort to restore knee stability, improve function, and limit long-term degeneration.^1^ While autografts are most commonly used to reconstruct the ACL, allografts have several potential advantages.^2^ Lack of donor-site morbidity, shorter operative time, decreased pain, improved cosmetics, and known graft size are often cited as their benefits.

Allografts have proven clinical success in ACL reconstruction but, as it relates to the allograft, can vary widely depending on the tissue type used, graft processing and preparation, and donor parameters.^3^^,^^4^ The age of the donor is one that is commonly thought to play a role. It is often felt that the younger the donor, the better quality of the graft and therefore decreased failure.

Most allograft tissue suppliers offer tendons ranging from 15 to 65 years in age.^5^^,^^6^ The availability of young donors is frequently limited and can delay surgery if the surgeon demands grafts of a set age limit. However, in vitro studies using double-looped tibialis tendon allograft tendons from specimens even older than 65 years of age have been shown to demonstrate comparable biomechanical strength and stiffness for tibialis tendons when compared with reported historical data for other ACL graft sources.^7^ Despite the positive basic science evidence, many surgeons will not change the donor age request because of a deficiency in clinical data. The lack of clinical evidence, along with the need of a more abundant tissue supply, prompted the study.

The purpose of this study is to evaluate the effect of graft donor age on outcomes of ACL reconstruction with non-irradiated, fresh-frozen allografts. Our hypothesis was that older donor age would not have a significant effect on clinical outcomes for ACL reconstructions.

## Methods

### Study Design

This prospective, randomized, double-blind, single-center study was approved by a central institutional review board (Western Institutional Review Board). Informed consent was obtained from all participants before enrollment. The study aimed to enroll patients aged 18-60 years with a unilateral ACL injury scheduled to undergo ACL reconstruction with a tibialis allograft and to follow them for 2 years postoperatively. Patients with meniscal injuries were included but excluded if additional pathology was present. Any previous knee surgery or history of injury to the contralateral knee were exclusions. Patients also were excluded if they were unable to comply with study requirements or had any systemic diseases or conditions that would potentially impact overall health, such as diabetes mellitus. Two groups of tendon donor ages were used for analysis: 18-50 years of age (Group A) and between 50 and 70 years old (Group B). Grafts were not preassigned to patients, as per the study design of graft donor ages ranging from 18 to 70 years with the following number of allografts for each decade: 1:10-19 years, 3:20-29 years, 8:30-39 years, 5:40-49 years, 8:50-59 years, and 12:60-69 years. The grafts were delivered by the processing company labeled A or B in no set order; the surgeon and patients were blinded to graft donor age.

### Surgical Procedure

The same surgeon and surgical technique were used for all patients. All ACL reconstructions were performed using the BioCleanse tibialis allograft (RTI Biologics, Inc., Alachua, FL). The grafts were processed through a validated system for viral inactivation and sterilization in accordance with Food and Drug Administration guidance, sterile packed, frozen, and stored at –40°C until the time of the surgical procedure. After thawing, the grafts were doubled over to a length of 8 cm and a diameter of 9 to 9.5 mm. The grafts were then placed under 20 N of tension until implanted. The operative technique was similar in all cases. An arthroscopic anteromedial portal approach was used for drilling of an anatomic placement of the femoral tunnel. The tibia tunnel was drilled thru a 1- to 2-cm incision at a 55° angle centered between the tibia spines and in line with the posterior aspect of the anterior horn of the lateral meniscus. Suspensory button fixation device was used on the femur and bioscrews on the tibia in all cases. Partial menisectomy or repair were performed if warranted.

### Rehabilitation

The standard of care rehabilitation protocol comprised 5 phases. The preoperative phase and early postoperative phase (0-4 weeks) focused on control of swelling and regaining range of motion. Patients were able to be weight-bearing as tolerated after surgery. Crutches were discontinued when the patient demonstrated normal gait. The intermediate postoperative phase (1-3 months) included exercises, stretches, and proprioceptive neuromuscular facilitation aimed at gradual strengthening of the knee and involved extremity to further regain function. The progressive conditioning phase (3-4 months) was aimed to further increase strength, endurance, and patient confidence in the knee. The sport-specific training phase (4 months and beyond) included exercises specific to the patient’s demands. Unrestricted return to sports and activities was only allowed once the patient had greater than 80% of the contralateral leg strength and passed functional testing. The time to full activities was a minimum of 6 months and more commonly 8 to 9 months.

### Outcome Assessment

The primary outcome measure was the International Knee Documentation Committee (IKDC) objective examination rating. Secondary outcome measures were subjective quality of life scores, including IKDC subjective scores and Lysholm knee scores.^8^^,^^9^ Patients were evaluated by the operating surgeon postoperatively at 10 to 14 days and at 1, 2, 4, 6, 12, and 24 months. The first postoperative evaluations included a radiograph as a baseline for tunnel position and fixation, and review of any adverse events. Subsequent follow-up visits included review of adverse events along with the IKDC objective examination and subjective quality of life scores described previously. A telephone contact was also made at 18 months postoperatively to review the patient’s health and knee function. Physical therapy assessment forms were completed at the 2-, 4-, and 6-month visits.

### Statistical Analysis

Pre- and postoperative scores were compared, and correlations were made between the donor age and the test scores. Before calculating the correlation coefficients, the Tukey method was used to identify outliers; no outliers were removed from the dataset. Correlational analyses were used to examine the relationship between donor age and clinical outcomes. In testing the null hypothesis that there is no difference in scores based on tendon donor age, an alpha level of 0.05 was used.

## Results

A total of 43 patients were screened with 40 enrolled participants that underwent ACL reconstruction with the study’s allografts. There were 2 screened patients who were not enrolled in the study due to having significant arthritic joint findings that precluded them from enrollment. One patient was precluded from the statistical analysis due to not disclosing a significant systemic disease until after surgery. Of the remaining 40 patients enrolled in the study, three patients were lost to follow-up and not included in the final data analysis. The remaining 37 patients (92.5%; Group A = 17, Group B = 20) were included in the final data analysis and followed for a minimum of 20.2 months and mean of 24.1 months (range 20.2-26.2). Patient demographics and details of ACL pathology at baseline are summarized in Table 1 and Table 2, respectively.

**Table 1:** Baseline Demographics

	Group A (n = 17)	Group B (n = 20)	*P* Value
Patient age, y, mean ± SD	42.1 ± 9.4	41.7 ± 8.5	.875
Graft donor age, y, mean ± SD (range)	33.8 ± 8.1 (18-46)	60.7 ± 4.8 (53-68)	<.001
Sex			.138
Female	14 (82.4%)	12 (60.0%)	
Male	3 (17.6%)	8 (40.0%)	

SD, standard deviation.

**Table 2:** ACL Pathology at Baseline

Test	Group A (n = 17)		Group B (n = 20)		*P* Value
	N	Percent	N	Percent	
IKDC Objective					.408
A	0	0.0	0	0.0	
B	1	5.9	0	0.0	
C	16	94.1	18	90.0	
D	0	0.0	1	5.0	
ND	0	0.0	1	5.0	

	N	Mean (SD)	N	Mean (SD)	
IKDC Subjective	17	39.2 (15.9)	20	39.4 (17.8)	.982
Lysholm	17	52.4 (19.2)	20	55.1 (18.9)	.675

ACL, anterior cruciate ligament; IKDC, International Knee Documentation Committee; ND, not determined; SD, standard deviation.

### Clinical Outcomes

The clinical outcomes at 2-year follow-up are reported in Table 3. Correlation between donor age and outcomes for the IKDC objective ratings (*P* = .56), IKDC subjective scores (*P* = .52), KT-1000 (*P* = 0.16), and Lysholm (*P* =0.44) indicated that there was no statistically significant association between clinical outcomes and age of the graft donor.

**Table 3:** ACL Pathology at 2-Year Follow-Up

Test	Group A (n = 17)		Group B (n = 20)		*P* Value
	N	Percent	N	Percent	
IKDC Objective					.45
A	15	88.2	19	95.0	
B	2	11.8	1	5.0	
C	0	0.0	0	0.0	
D	0	0.0	0	0.0	
KT-1000					.28
0	4	23.5	2	10.0	
1	10	58.8	10	50.0	
2	2	11.8	6	30.0	
ND	1	5.9	2	10.0	

	N	Mean (SD)	N	Mean (SD)	
IKDC Subjective	17	86.1 (16.2)	20	84.1 (15.6)	.70
Lysholm	17	91.4 (16.7)	20	88.1 (12.3)	.49

ACL, anterior cruciate ligament; IKDC, International Knee Documentation Committee; ND, not determined; SD, standard deviation.

### Adverse Events

No additional surgery was required during the initial 2-year follow up. Although outside of the study time limits, it can be anecdotally noted that one allograft from Group A tore from trauma at 44 months postoperatively as noted during consultation with the study’s surgeon. Additionally, it can be noted 2 medial meniscus repairs tore, one at 29 months (Group A) and the other at 35 months (Group B).

## Discussion

The main finding from this prospective, randomized study is that the data did not show a difference in patient reporting outcomes at the 2-year follow up in patients who underwent ACL reconstruction with tibialis allografts from donors >50 years of age compared with donors <50 years of age. This is a clinically relevant finding because many surgeons may be hesitant to use grafts from older donors, and supply from young donors is at times insufficient to meet demand.

The use of allograft tissue for ACL reconstruction encompassing all age groups is not uncommon, with a survey by the American Academy of Orthopedic Society for Sports Medicine of 833 surgeons reporting allografts use in 27% of primary ACL reconstructions and in one large hospital system registry of 21,304 ACL reconstructions allografts were used in 41% of the cases.^10^^,^^11^ Of no surprise, the tissue availability can be inadequate with the concern of donor age by some surgeons being a contributing factor.

ACL reconstruction with allograft tissue is reported to have differing success in the orthopedic literature. It has been well documented that patients age of 25 or younger with high activity levels are at greatest risk for failure.^12^^,^^13^ While there is general consensus that allografts should not routinely be used in patients of this category, studies have shown allografts can have comparable results to autografts.^14^, ^15^, ^16^, ^17^, ^18^

Other variables need to be taken into consideration before evaluating only the donor age. The graft itself should be from an American Association of Tissue Banks–certified tissue bank to assure proper screening and procurement. The processing method and inherent graft strength can also have a role in outcome beyond the age of the graft.^19^ As none of the key outcomes met statistical significance when comparing across groups, a retrospective sample size and power analysis were performed assuming existing effect sizes and distributions. Both the IKDC subjective and Lysholm outcomes were well short of a *P* value of .05, and hence the expected sample size for significance is correspondingly large: 974 for the former and 306 for the latter. Similarly, the IKDC objective assessment showed a needed sample size of 259, whereas the KT-1000, which was closest to significance in the existing data, would have required a sample size of 85, the smallest of the four by a considerable margin.

From another perspective, at the actual sample sizes available, retrospective power for detecting a significant difference between groups was 7% for the IKDC subjective, 10% for Lysholm, 11% for IKDC objective, and 31% for the KT-1000. Available power was therefore low for all outcomes.

Although aseptic technique can limit the risk of infection, tissue banks routinely use sterilization techniques, which can have significant effects on tissue strength. Irradiation is most commonly used, but levels ≥25 Mrads lead to tissue deterioration, which can affect clinical outcomes.^20^^,^^21^ Even doses as low as 1.2 Mrads can decrease graft stiffness by 20%.^22^ Chemical processing can be performed, but many such as ethylene oxide have been discontinued.^23^ BioCleanse is one chemical-processing method that has been reported to have no detrimental effects on preimplantation biomechanical properties strength and used in this study.^24^^,^^25^

The tibialis tendon allografts procured by an AATB-certified tissue bank and processed in a single method. While one cannot state that tibialis tendons are clinically better simply based on biomechanical testing, their ultimate load to failure is as high or higher than other grafts before implantation. In a systematic review by Lansdown et al.^26^ examining common ACL grafts, the native ACL is reported to have a load to failure of 1,730 to 2,160 N, whereas a double looped tibialis tendon was greatest at 3,012 to 4,112 N.

Several basic science studies have looked at the mechanical properties relating to graft donor age, but results vary because different grafts, processing, and testing methods were used. However, the general consensus on critical analysis of the studies with stringent parameters is that grafts from donor age ≤65 years (upper limit of most tissue bank suppliers) have limited differences in biomechanical properties.

Greaves et al.^27^ tested 126 tibialis tendon allografts divided into 3 age groups: <45 years, 46-55 years, and 56-65 years. They found that donor age up to 65 years did not significantly affect the initial failure load, stiffness, or displacement of failure. Jones et al.^28^ tested BioCleanse versus untreated bone–patellar tendon–bone (BPTB) grafts. Although they found no difference in biomechanical properties between the processed and unprocessed grafts, they also found no significant differences between donor grafts 29-40 years old compared with 41-65 years of age. However, the grafts in the 65-90 years age group did have significantly less maximum strength. Blevins et al.^29^ tested 82 BPTB procured from donors aged 17-54 years of age and found no significant correlation between tensile strength and donor age.

In the largest number of allografts tested Swank et al.^30^ reported their results of 550 tibialis posterior tendons. The specimens were divided into 6 donor age groups: 15-29, 30-39, 40-49, 50-59, 60-69, and 70-79 years and underwent numerous tests including stiffness, ultimate tensile force, strength and displacement. They found that ultimate tensile strength increased slightly with age up to 40-49 years and decreased with further increase in age. However, the magnitude of difference between age groups was <15% for all outcome parameters. Their conclusion was that age explained at most 6% of the variation in structural and mechanical properties of tibialis posterior allograft tendons, and most likely not clinically relevant.

Although well-controlled basic science studies indicate that the donor age should not have a significant effect on graft failure rate, a concern of its clinical application exists because of lack of clinical data. Only a few studies have specifically evaluated outcomes as it relates to donor age.

Hampton et al.^31^ reported in a retrospective study on the outcome of 75 patients at a mean 2-year follow-up after BPTB allograft. Donor age ranged from 14-65 years and the average patient age was 37 years (range 18-60 years). The finding was that the donor age had no effect on the degree of postoperative improvement using Lysholm or Tegner scores. However, information was gathered only via phone calls and chart review with no objection assessment performed.

In a retrospective cohort study of 5968 primary ACLR with allograft tissue, Tejwani et al.^32^ reported on the revision rates looking at several variables. The donor age was separated into 4 age groups: <20, 20-40, 41-59, and >60 years with the number of cases in each group being 276, 1,301, 3,497, and 892, respectively with 2 not reported. The revision rate was low for all groups with the percent being 2.17, 2.84, 2.49, and 2.91 in chronologic order of young to old. They concluded that the donor age did not significantly affect revision risk.

The data collected were extensive, and the revision rate across all donor age categories was similar. Numerous other variables were included at the same time as looking at donor age. The grafts ranged from BPTB (17.2%), Achilles tendon (19.9%), to all soft tissue (62.9%). Four different sterilization methods were used, including 1,146 (19.2%) had irradiation of >1.8 Mrads. as negative effects on the graft strengths as cited previously. In addition, the age of the patients varied widely with 1,747 (29.3%) being in the high-risk age group of ≤25 years.

Shumborski et al.^5^ reported differing results when it came to the age of the allografts and outcomes. They reported that tendons from female donors >50 years had significantly greater rates of graft failure compared to male donors aged <50 years. However, they used multiple tissues for grafts including patellar tendon, Achilles tendon, and tibialis. When they analyzed the outcome based solely on graft type, they found the failure rate was 11% for tibialis, which we studied, and combined failure of 29.9% for patellar tendon and Achilles tendon. The most critical factor in comparing their study to this one is the patient’s age which is known to have an increased failure rate. Patients in their cohort were younger than the age of 25 years with a range of 13 to 25 years.

In this study, it demonstrated that donor age was not associated with tibialis tendon allograft failure. Although the donor age groups were separated into younger than older than 50 years of age, the groups were noticeably more divided in age. The younger group donor average was 33.8 years, with the oldest being 46 years. The older group average was 60.7 years, with the youngest being 53 years. The number of patients was small, but it had the benefit of being prospective and randomized with a single surgical technique and all other graft variables controlled to allow specific evaluation of donor age. In addition, both subjective and extensive objective evaluations were performed.

As none of the key outcomes met statistical significance when comparing across groups, a retrospective sample size and power analysis were performed assuming existing effect sizes and distributions. Both the IKDC subjective and Lysholm outcomes were well short of a *P*-value of .05, and hence the expected sample size for significance is correspondingly large: 974 for the former and 306 for the latter. Similarly, the IKDC objective assessment showed a needed sample size of 259, whereas the KT-1000, which was closest to significance in the existing data, would have required a sample size of 85, the smallest of the four by a considerable margin.

From another perspective, at the actual sample sizes available, retrospective power for detecting a significant difference between groups was 7% for the IKDC subjective, 10% for Lysholm, 11% for IKDC objective, and 31% for the KT-1000. Available power was therefore low for all outcomes. Longer follow-up will be needed to see if the results of this study will be upheld.

### Limitations

This study is not without limitations. The main limitation of this study was the small patient population, which was not large enough to show statistical significance. In standard practice, patients rarely ask the age of the donor. However, the number of patients to agree to participate in a prospective, randomized study using an allograft of a wide donor age group was limited and thus accounted for the study size. The protocol was to enroll 50 patients, but we were only able to enroll 40 patients over a 6-year time period. The age of the graft donors affected enrollment as potential patients were apprehensive to receive an older donor tendon which led to enrollment being stopped prior to the initial goal of 50 patients.

An additional limitation was the predominant sex in both groups was female. Further, Group A had a greater proportion of females to males (82.4%) compared with Group B (60%). The composition of the groups was solely on the randomized order of presentation and thus could not be controlled in our study design.

## Conclusions

Donor age was not associated with clinical outcomes after ACL reconstruction using nonirradiated, fresh-frozen tibialis tendon allografts.

## Supplementary Data

ICMJE author disclosure forms
